# Comparison of the Latin America Regulation Landscape and International Reference Health Authorities to Hasten Drug Registration and Clinical Research Applications

**DOI:** 10.1007/s43441-023-00565-7

**Published:** 2023-09-08

**Authors:** Urimara Argotti, Lada Leyens, Carlos Lisbona, Pilar López, Sergio Alonso-Orgaz, Angel Nevado, Virginia Cozzi

**Affiliations:** 1 International Regulatory Policy Department, Latin America Productos Roche, S.A. de C.V., Mexico City, Mexico; 2 Product Development Regulatory, F. Hoffmann-La Roche AG, Basel, Switzerland; 3 Medical Affairs Department, Roche Central America, Venezuela, and the Caribbean, Heredia, Costa Rica; 4 Medical Writing Department, LIDESEC S.L, Madrid, Spain

**Keywords:** Latin America regulations, Marketing authorization procedures, Innovative clinical trial designs, Expedited regulatory pathways, Reliance pathways, New clinical evidence approach

## Abstract

**Introduction:**

Promptly providing new drugs to fulfill unmet medical needs requires changes in drug development and registration processes. Health Authorities (HAs) considered as reference due to their experience and acknowledgement (Food and Drug Administration [FDA] among others) already consider innovative clinical trial (CT) designs and flexible approval procedures, but Latin America (LATAM) regulations are still far. A comparison was performed to identify gaps.

**Materials and Methods:**

CT requirements for drug Marketing Authorization Application (MAA) and CT approval regulations were compared between LATAM and reference HAs (FDA/European Medicines Agency [EMA]/Health-Canada/Swissmedic/Therapeutic Goods Administration [TGA]/Pharmaceuticals and Medical Devices Agency [PMDA]), as of August 2022. Procedure included reference HAs regulations review, item selection, identification in LATAM regulations, and International Council for Harmonisation of Technical Requirements for Pharmaceuticals for Human Use (ICH) guidelines (ICH-E6[R2]/ICH-E8[R1]) implementation revision.

**Results:**

For MAA, specific application requirements or ICH guideline M4(R4) on common technical document (CTD) adoption are generally stated, and phase-I/III performance is mandatory (explicitly/implicitly). Faster patient access procedures are infrequent: Priority-drug programs, conditional authorizations, or expedited procedures are scarce or non-existent. Regulatory reliance procedures are adopted through different pathways. Regarding CT approval, innovative/complex CT designs are not prohibited but usually omitted. Some countries implemented adapted CT conducting during the COVID-19 pandemic. Early scientific advice meetings (HA-sponsor) are occasionally considered. Most countries are not formally ICH-joined.

**Conclusions:**

LATAM regulations must adapt to new regulatory standards (FDA/EMA/ICH) through implementation of frequent updates, reliance/expedited procedures, early HA-sponsor interactions, innovative/complex CTs, mandatory phase-III reaching elimination, and decentralized elements for CT conducting.

## Introduction

The need to provide innovative drugs promptly to patients to fulfill unmet medical needs is changing the drug development scene. It has been demonstrated that the traditional approach to evidence generation poses challenges to achieving the necessary efficient development that could lead to rapid drug approval [1, 2]. In most countries, the necessary change is hindered at marketing and pre-marketing authorization levels: Requirements for new drug/indication approval still entail data obtained from the traditional phase I–III studies, and CT authorization regulations do not always consider new CT designs.

Advances in research methodologies and new technologies offer tools to implement innovative and efficient ways to investigate drugs [3, 4], fostering their availability faster [4].

New CT approaches include enrichment designs [5], adaptive designs [6], master protocols (umbrella, basket, or platform designs) [5], and use of historical controls [5].

These strategies are already being implemented and accepted, by the Food and Drug Administration (FDA), for authorization of new treatments in many pathologies, especially in oncology [7, 8]. In this sense, a survey performed in 32 pharmaceutical companies [9] revealed that 66% had conducted innovative CTs to provide evidence for many purposes, including for first drug approval in 59% of the companies.

Some Health Authorities (HAs), considered as reference due to their experience and international acknowledgement, through continuous updating, have taken actions (guidelines and initiatives) to be open to evidence coming from innovative CTs. The FDA led this change, beginning in 2004 (“*Critical Path Initiative for transforming development, evaluation, and manufacturing of medical products”*) [10] and continuing afterward (in 2018, *“Complex Innovative Trial Designs Pilot Meeting Program”*) [5]. The European Medicines Agency (EMA) started in 2007 (*“Reflection Paper on Methodological Issues in Confirmatory Clinical Trials Planned with an Adaptive Design”*) [10] and also carried out later actions (in 2019, *“Recommendation Paper on the Initiation and Conduct of Complex Clinical Trials”*) [11]. Health Canada and Medicines and Healthcare products Regulatory Agency (United Kingdom) consulted with stakeholders in 2020 and 2021, respectively, for the modernization of CT regulatory frameworks [12, 13]. Relevant questions were considered such as a more agile life cycle approach, including new types of innovative CTs and improving timelines, a risk-based approach, the performance of decentralized CTs, modernization of compliance and enforcement, collaboration across sectors, education on regulatory science, or the use of real-world data, among others [12, 13].

Reference HAs have also established alternative pathways to the traditional Marketing Authorization Application (MAA), such as priority-drug programs [14], expedited review [15], conditional approval [15], and reliance pathway [16].

In the case of Latin America (LATAM) countries and those Caribbean islands with regulations (Argentina, Aruba, Bolivia, Brazil, Chile, Colombia, Costa Rica, Cuba, Curacao, Dominican Republic, Ecuador, El Salvador, Guatemala, Guyana, Honduras, Jamaica, Mexico, Nicaragua, Panama, Paraguay, Peru, Sint Maarten, Trinidad and Tobago, Uruguay, and Venezuela), for MAA and traditional CT authorization, these are not as exhaustively described as those in other countries. Therefore, a new drug/indication may find delays, or even no chance, of being available to patients due to either a specific prohibitive regulation or the lack of flexibility in the regulation. On the other hand, some procedures and initiatives to overcome these challenges are implemented, albeit with differences across LATAM countries [17, 18]. However, there is no available analysis of the whole landscape that could help us to undertake accurate actions in each country to speed drug marketing approval.

Our primary objective was to evaluate the situation of the regulations for drug registration in relation to the CT requirements of LATAM. This was compared with those of the main reference HAs to identify gaps that hinder rapid drug development and propose recommendations to close them.

## Materials and Methods

To evaluate MAA requirements and CT approval regulations, LATAM countries and those Caribbean islands with regulations, described in the Introduction section, were chosen. Reference HAs selected were FDA, EMA, Health Canada, Swissmedic, Therapeutic Goods Administration (TGA) in Australia, and the Pharmaceuticals and Medical Devices Agency (PMDA) in Japan.

Reference HAs and LATAM regulations were reviewed by examining the contents published on their corresponding websites. A comprehensive search was done in each country through an initial review followed by an in-depth one when contents of interest were not found initially.

Relevant items (key concepts) to address the gaps, selected by analyzing the reference HA regulations, corresponded to different steps in drug development and belonged to two main categories: (1) MAA and (2) CT authorization. Key concepts for each category and their descriptions considered for the purpose of this evaluation are detailed in Table 1.

**Table 1:** Key concepts selected for the analysis of Latin America regulations

Key concept	Description
(1) MAA	
(a) Standard	MAA follows the usual procedure with no exception regarding requirements or timelines for evaluation
(b) For generics	MAA for drugs produced to be equal to an already marketed drug, based on the efficacy and safety of the marketed drug to get their authorization
(c) For faster patient access	
(I) Priority drug programs	Programs designed to facilitate the development and expedite the review of drugs that treat serious conditions and fill an unmet medical need based on promising animal or human data. By means of these programs, important new drugs are available to the patient earlier [19]
(II) Expedited review	Regulatory authorities speed the review of certain products to enable faster approval. The review time of an expedited review is substantially shorter than the review time of a standard review [34]
(III) Conditional approval	Approval based on preliminary data of drugs generally aimed at diseases with a significant impact on morbidity and/or mortality but lacks adequate treatment options and requires additional data to be converted into a “full approval.” If additional data do not confirm the earlier promise of benefit, approval may be withdrawn [15]
(IV) Reliance	Act whereby the National Regulatory Authority in one jurisdiction may take into account and give significant weight to – i.e., totally or partially rely upon – evaluations performed by a stringent regulatory authority in reaching its own decision. The relying authority remains responsible and accountable for decisions taken, even when it relies on the decisions and information of others [34]
(2) CT authorization application	
(a) CT /phase definitions	Descriptions of what a CT is and the stages for clinical drug development
(b) CT application	
(I) Standard	CT application following the usual requirements set out by a HA
(II) Reliance for CT approval	CT application following the definition of reliance as described above
(c) Special considerations	
(I) COVID-19	Regulations aimed at CTs developed during COVID-19 pandemic
(II) Women	Specific considerations regarding the participation of women in CTs
(III) Cell therapy	Requirements to carry out CTs in cell therapy
(IV) Indigenous	Indications for the participation of specific communities with their own culture and traditions inside a country
(d) Scientific advice meetings	Meetings where sponsors receive scientific advice from HAs about the most appropriate way to generate robust evidence on a medicine`s benefits and risks [35]
(e) Complex innovative trial design	CT design that differs from the standard randomized controlled trial design and delivers results more efficiently, reduces the study timeline, and maximizes the knowledge gained. Common examples include umbrella, basket, adaptive, platform, dose-ranging, targeted or stratified, Real-world, and Bayesian studies [36]
(I) Master protocols	Unifying study construct that includes multiple subgroups and substudies, with patients having the same or different diseases and that employ one or multiple drugs to treat it [37]
(II) Adaptive design	Design that allows modifications to the trial and/or statistical procedures of the trial after its initiation without undermining its validity and integrity [5]
(III) Enrichment strategies	Designs intended to increase the efficiency of drug development and support precision medicine by tailoring treatments to those patients who will benefit based on clinical, laboratory, genomic, and proteomic factors [5]
(IV) Decentralized CTs	CTs taking place remotely, without a physical visit to a trial site, after the necessary technology has been installed and explained to the patient [12]

*COVID-19* Coronavirus disease 2019, *CT* clinical trial, *HA* health authority, *MAA* marketing approval application

In addition, we reviewed the implementation of two International Council for Harmonisation of Technical Requirements for Pharmaceuticals for Human Use (ICH) guidelines belonging to the efficacy category: E6-Good Clinical Practice (R2) (ICH E6 [R2]) and E8-General Considerations for Clinical Trials (R1) (ICH E8 [R1]).

Information regarding key concepts from LATAM and reference HAs was searched as of August 2022 and organized into tables. Next, gaps and relevant differences with respect to reference HAs were identified. Finally, actions were proposed to improve marketing approval and CT development requirements in LATAM countries.

**Figure 1:**
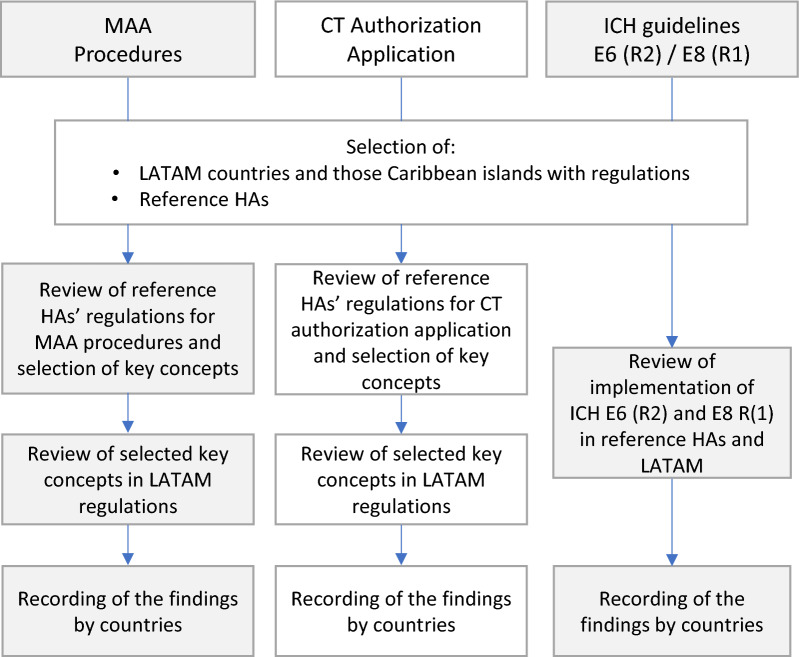
Data analysis procedure for the selected key concepts corresponding to Marketing Authorization Application (MAA), Clinical Trial (CT) Authorization Application and ICH guidelines E6 (R2) and E8 (R1).

Figure 1 shows a diagram with our data analysis procedure.

## Results

The findings on the regulations for each process (MAA and CT authorization) are presented separately below.

### MAA Procedures

Overall, while most of the key concepts selected to assess the MAA requirements were found in reference HAs, especially FDA and EMA (Table 2), LATAM regulations frequently lacked the corresponding items. This absence is remarkable for the MAA procedures that intend to achieve faster patient access to drugs (except reliance pathways).

**Table 2:**
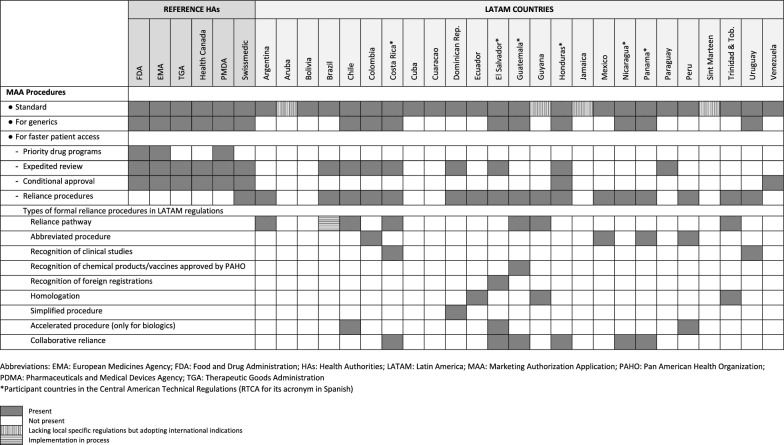
Key concepts on marketing authorization application procedures identified in reference health authorities and Latin America regulations

Unless otherwise stated, the reviewed regulations apply to chemical and biological products.

Standard MAA requirements are described more exhaustively in reference HAs than LATAM regulations, where the extent of contents can vary significantly among countries. Four of twenty-five countries assessed in LATAM have no local regulation, so they adopt international indications such as the “ICH guideline M4 (R4) on common technical document (CTD) for the registration of pharmaceuticals for human use—organization of CTD Module 2. Common Technical Document Summaries”. The requirement of phase I-III study development to obtain drug approval is specified in Chile, Brazil, Ecuador, and Venezuela regulations and in countries adopting Central American Technical Regulations (RTCA for its acronym in Spanish), which are Costa Rica, El Salvador, Guatemala, Honduras, Nicaragua, and Panama.

For innovative biotechnological products, Guatemala considers an exemption of phase III studies in exceptional cases of early development of clinically relevant drugs if previously authorized by a WHO-classified reference HA. This is referred to that biotechnological product first marketing-authorized in the first origin country, supported by the complete quality, safety, efficacy, and immunogenicity documentation, being the reference product. No specification for the required phases was found in 8 of 25 LATAM countries. However, when searching information regarding CT conducting before drug commercialization, these regulations only mention the traditional phase I–III development, apart from Cuba, where the possibility of having overlapped phases (i.e., a phase II/III CT) is considered.

In many cases, a separate section is found for biological drugs, globally defined as those of which obtaining and/or production involves living organisms, as well as their fluids or tissues.

An abbreviated procedure for new drug application that is a generic/bioequivalent drug of authorized one is implemented in all reference HAs. However, in LATAM regulations, this process is only included in Chile (with a specific mention for biotechnological products), Colombia and countries adopting RTCA, besides, Guatemala has its own regulation.

In general, procedures for faster patient access to new drugs can be grouped into four classes: programs for priority drugs, expedited review, conditional approval, and reliance pathways.

Programs for priority drugs are focused on drugs aimed to cover unmet needs in serious or life-threatening diseases and directed to provide faster development and evaluation for earlier availability to patients. They are named differently by each HA. These programs are only implemented in FDA (Fast-track), EMA (Priority medicines [PRIME]), and PMDA (Strategy of SAKIGAKE). They are not present in the rest of the reference HAs or LATAM regulations.

Regarding expedited review, which implies procedures that shorten the time for evaluation of the MAA for innovative drugs of interest for public health, it receives distinct designations across regulations. All reference HAs have implemented this process with different features (priority review in FDA, TGA, Health Canada, and Japan, accelerated assessment in EMA, and fast-track procedure in Swissmedic). In LATAM regulations, they are considered in some countries, as shown in Table 2. Brazilian normative establishes that for the registration of a new drug aimed to prevent or treat serious diseases being life-threatening or heavily debilitating, if it fulfills an unmet medical need, phase-III CTs are not necessarily to be ended, or even only ended phase-II CTs are required if phase-III CTs are not applicable (also for biologicals). There is an expedited procedure in Chile, since 2020, for chemical drugs, and is planned to be widened to biological products under certain circumstances. In the case of Colombia, authorities prolonged for 6 months the urgency approval procedure for COVID-19 drugs.

Conditional approval is granted for drugs treating serious or life-threatening diseases that provide meaningful therapeutic benefit over existing treatments, for which promising data from phase I–II CTs or from surrogate endpoints are obtained. It implies an engagement by the marketing authorization holder to comply with a post-marketing plan to develop confirmatory studies and evaluation of safety issues to obtain the “full approval”. This procedure is found under different denominations among regulations. All reference HAs have included this procedure. In LATAM countries, this procedure was found in Venezuela (for extendable 6-month periods) and Honduras (6-month permission for imported drugs) only.

Regulatory reliance is not implemented by reference HAs, except Swissmedic. However, some LATAM countries consider diverse reliance pathways (see Table 2). Those presenting an official procedure are Argentina, Chile, Colombia, Costa Rica, Dominican Republic, Ecuador, El Salvador, Guatemala, Honduras, Mexico, Nicaragua, Panama, Peru, and Uruguay. A collaborative reliance (Joint Mechanism for the Evaluation of drug product files in Central America), not yet for biologicals, among countries was recently implemented in Costa Rica, El Salvador, Guatemala, Honduras, Nicaragua, and Panama (the two last ones are not actively involved). Colombia and Peru mention the reliance on adoption in their regulation; however, there have been very few examples in practice. In the case of Ecuador, the current process does not represent a better timeline for regulatory evaluation. The National Health Surveillance Agency (ANVISA for its acronym in Portuguese) in Brazil is still evaluating the comments from an industry survey before implementing a formal procedure. In addition, reliance is also applied by the Caribbean Regulatory System (CRS), being currently adopted by Guyana and Trinidad and Tobago only.

### CT Authorization Application

Generally, most of the key concepts selected to assess CT approval regulations were found in reference HAs, especially FDA and EMA (Table 3), however LATAM regulations frequently lacked the corresponding concepts, being striking for complex, innovative CT designs (Table 3).

**Table 3:**
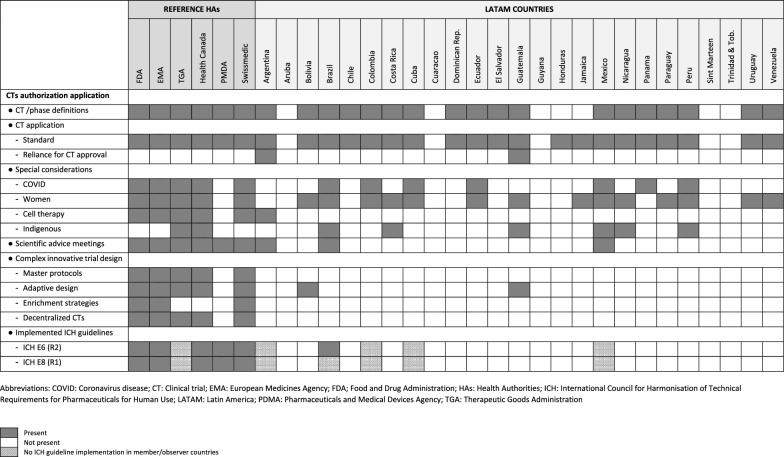
Key concepts on clinical trial authorization application identified in reference health authorities and Latin America regulations

With respect to CT approval requirements, the definition of CT and investigational phases are described in reference regulations. Most LATAM ones include CT (or synonymous term) definition. This was not found in countries with low performance of CTs (Aruba, Curacao, Guyana, Jamaica, Sint Maarten, and Trinidad and Tobago). In the case of Honduras, although there is CT normative, no definition of such activity is included in its regulations.

In the countries that describe the type of CT and its phases, they refer to the standard phases (I, II, III, and IV). Some regulations containing CT definition do not include the description of the phases, such as Dominican Republic, El Salvador, and Panama. Generally, in both LATAM and reference regulations, phases I, II, III, and IV are described following the traditional concepts of drug development. However, when defining CT in Cuba, they consider the possibility of overlapped phases (i.e., a phase II/III CT). In the case of phase III, it is usually defined as the phase providing the needed confirmatory information for the studied indication, and even mention of its support for marketing authorization is clearly stated in some regulations.

Requirements for CT application (standard procedure) are described in reference and almost all LATAM regulations, as shown in Table 3. In the case of Health Canada, although not yet approved, a proposal to make the procedure more flexible is in progress. Overall, there is no explicit prohibition to carry out CTs with innovative designs. However, in some LATAM countries (Argentina, Chile, Colombia, Dominican Republic, Ecuador, El Salvador, Honduras, and Mexico) some type of specification about traditional phases (I–IV) are described, so it could be understood that only standard CT designs are considered under those regulations. In Cuba, as explained above, CTs with overlapped phases are possible.

As an alternative procedure to accelerate CT approval, the reliance pathway based on previous authorization by other HA is considered in Argentina and Guatemala, although is not included by reference HAs.

During COVID-19 pandemic, flexible procedures to facilitate CT conduct were set by most reference HAs and some LATAM countries (see Table 3).

Regulations for the innovative cell therapy CTs were found in most of reference regulations, but only in Argentina for LATAM countries, being less exhaustive, specially vs. FDA, EMA, or Health Canada, although the main principles are considered equally.

All reference HAs have set scientific advice meetings with sponsors, in some steps of the drug development, to contribute to the earlier access of the patient to new drugs and to foster more effective drug development by improving scientific resource utilization and enhancing patients’ participation. In the EMA, early advice is offered via the Innovation Task Force meeting. This type of meeting is only considered in Argentina, Brazil, and Mexico for LATAM countries.

With respect to complex innovative trial designs, master protocols (umbrella, basket, and complex trials with features of both) are described in a guidance for industry in FDA, being a part of the category of complex CTs. EMA and Swissmedic consider master protocols by relying on the recommendation paper of the Clinical Trial Facilitation and Coordination Group (CTFG). Health Canada consulted stakeholders in 2021 about the modernization of CTs, where this concept was treated (including its implementation for pediatric studies), but the results of the consult have not been implemented yet. TGA also considers these concepts in its regulations. No corresponding information was found for PMDA. None of LATAM countries have implemented normative for master protocols.

Adaptive designs are considered by most reference HAs (see Table 3). Only in Guatemala and Bolivia the term “sequential clinical trial” is found, where the final sample size depends on the intermediate results of the trial. No other related information is found in the rest of the LATAM regulations.

Enrichment designs are described in FDA regulations. The EMA and Swissmedic rely on the CTFG recommendation paper and the Q&A on complex clinical trials from EMA. Other reference HAs and LATAM countries do not include this strategy.

Aspects about decentralized CTs that use digital health technologies for remote data acquisition are present in almost all reference HAs, as shown in Table 3. However, this concept is not found in LATAM regulations.

Regarding the adoption of ICH E6 (R2) and ICH E8 (R1) (Table 3), reference HAs, all ICH members except TGA (observer), have implemented, or are in the process of implementing, these guidelines. For LATAM countries, Brazil and Mexico are members, but these guidelines are only implemented in Brazil. Argentina, Colombia, and Cuba are observers. The rest of LATAM countries have not formally joined the ICH.

## Discussion

MAA and CT authorization regulations are aspects that must be changed to facilitate quicker and safe access of patients to new drugs.

Our review provides an overview of the current procedures for MAA and CT approval that are discussed below:

### MAA Procedures

Overall, patient access to new drugs is not as rapid as required to address unmet medical needs. The standard drug approval procedures currently established by many HAs, imply numerous and, sometimes, complex steps [15].

In this research work, first, we reviewed the **requirements for MAA**. All LATAM countries consider regulations regarding this concept, although some of them do not have specific local rules; therefore, they adopt international ICH guideline M4.

The **need for phase I–III development,** as a traditional requirement, is stated in some regulations (Chile, Brazil, Ecuador, and Venezuela), including those countries adopting RTCA where conclusive data of such CTs are required; Guatemala has an exemption of phase III studies for exceptional drugs of clinical relevance previously authorized by a WHO-classified reference HA. For the rest of countries having local regulations, no such specification appears but considering CTA requirements, some type of specification about traditional phases before drug registration is described. Therefore, it could be understood that they only consider traditional CT designs, apart from Cuba, where a flexible feature is found when defining the term “clinical trial” with the possibility of phase-combined studies.

The review of the **procedures to speed MAA** and provide faster patient access showed that there are programs for priority drugs that are promising to cover unmet medical needs. These programs shorten the drug development and the marketing approval for an indication and are included in three of the reference regulations (FDA, EMA, and PDMA). In the case of the FDA, once a drug receives “fast track” designation, early and frequent communication with the drug company is encouraged throughout the entire drug development and review process, and often leading to earlier drug approval and access by patients [19]. These types of programs, not considered by LATAM and the rest of the reference regulations, are difficult to implement, although they would be very effective as it reduces the time for drug development from early phases. Secondly, **expedited review,** which reduces the time for evaluating a MAA, such as the “priority review” program in FDA that leads to review in 6 months vs. 10 months for a standard review [20], is included in some LATAM regulations. Brazilian normative also considers that phase III studies do not necessarily need to be ended, or even only ended phase II studies are required when phase III CTs are not applicable, for the registration of a new drug aimed to prevent or treat serious diseases that are life-threatening or heavily debilitating if it fulfills an unmet medical need. A third way to accelerate access to new drugs is through **conditional approval,** which grants early authorization for drugs considered especially needed but requires further data obtained during post-marketing use to get the definitive approval. This is not usually accepted by LATAM HAs (with two exceptions: Venezuela and Honduras), in contrast to all reference regulations. Overall, these types of expedited procedures reduce the timelines for drug development when implemented by HAs. For example, the median development time for drugs in at least 1 expedited program was 7.1 years (interquartile range [IQR], 5.1–10.1) compared with 8.0 years for non-expedited drugs (IQR, 6.5–10.0; *P* = 0.04) [20].

In LATAM regulations, however, it is more common to find some type of **reliance procedure** to speed up the drug approval process. This mechanism is implemented through several ways across countries. Reliance in a formal manner, implying an immediate authorization if the drug is already approved by recognized HAs, is adopted by Argentina, Brazil (in progress), Chile, Costa Rica, Guyana, and Trinidad and Tobago. This pathway has the advantage of being quicker than other reliance modalities, such as the abbreviated procedure (Mexico and Panama), recognition of clinical studies (Costa Rica and Uruguay), recognition of chemical products and vaccines approved by PAHO (Guatemala), recognition of foreign registrations (El Salvador), homologation (Ecuador, Guyana, and Trinidad and Tobago), simplified procedure (Dominican Republic), accelerated procedure (only for biologics, in Chile, El Salvador, and Peru), or collaborative reliance (Costa Rica, El Salvador, Guatemala, Honduras, Nicaragua, and Panama). Countries implementing these types of reliance procedures could be more receptive to adopting a formal one, thereby facilitating quicker drug MAA approval. On the other hand, those countries that mention the reliance procedure in their regulations, but their implementation is not done (Colombia and Peru) or do not even shorten timelines (Ecuador) should modify their specifications to make this procedure real and effective. WHO supports the use of the reliance pathway with recommendations that HAs adopt these types of approaches that take into account decisions from reference agencies and evaluate only specific issues of local responsibility [21].

Currently, there are examples of drugs getting marketing approval by using innovative trial designs. Adaptive designs (including adaptations in formulation selection, new primary efficacy endpoint, dose selection, or sample size adjustment) and master protocols (basket, umbrella, and platform) have been used as part of the evidence dossier to get marketing approval from FDA and EMA [5, 10]. The different authorizations were granted for diseases including hematological malignancies and solid tumors (melanoma, breast cancer, urothelial carcinoma, among others), and included adult and pediatric populations. Some of them were accelerated or conditional approvals following a phase II trial [10, 22].

An example of an initiative that considers innovative CT designs and early alignment among stakeholders, including the FDA and the EMA, is ACCELERATE [23]. ACCELERATE aimed to accelerate the authorization of innovative treatments for children and adolescents with cancer so they can benefit rapidly and reduce the burden of further sequelae.

Although this review did not include the regulations about **real-world evidence** (RWE) use as a support in pre-marketing for decision-making, it is worth mentioning that this new approach, incorporated into regulatory processes by FDA and the EMA and by other regulatory bodies in an earlier stage [10], has not been found in LATAM regulations.

### CT Authorization Application

Traditional CTs, although being considered the paradigm of drug research, show issues that hamper the agile arrival of needed drugs to the market [1, 2].

Comparing LATAM and reference regulations, a relevant difference is observed with respect to **innovative CT design descriptions** (**master protocols, adaptive designs, enrichment strategies, or decentralized CTs**). Most reference regulations include these new approaches that allow faster patient access to new drugs [10, 24, 25] but most LATAM countries have not considered them in their rules or guidances for CT approval procedures. This means that, although innovative CT designs are not explicitly forbidden, usual CT designs and traditional phases (I-III) should still be followed before marketing approval is obtained in these countries. Currently, in the case that innovative designs could be considered during the development of any drug, their implementation is difficult or even not possible due to the lack of regulation. During the COVID pandemic, unusual methods to carry out CTs had to be implemented [26, 27], showing that more flexible procedures were safe and effective. Thus, it was demonstrated that it is possible to change standard designs without losing reliability in the clinical investigation. In fact, many reference HAs and some in LATAM countries (Brazil, Colombia, Cuba, Ecuador, Mexico, Panama, and Peru) developed rules for performing CTs during this period. Some of these measures, such as reducing the burden of administrative procedures, designing decentralized trials, or using electronic informed consent, would be aligned with modernization purposes.

Other measures to facilitate the development of a drug, as well as the performance of innovative CTs [5], would be carrying out **scientific advice meetings** between HAs and sponsors. These meetings happen at several points of the drug development lifecycle and are useful to align the objectives to reach for both parties from early stages. These meetings are described in reference regulations but only in very few LATAM countries (Argentina, Brazil, and Mexico).

Additional reference points are the revisions of applicable international guidelines to ensure the quality and data integrity of CTs**.** The **ICH E8 (CTs performance guidelines)** is modified by ICH E8 (R1) [28], which includes details about quality for CTs, stakeholder engagement, CT design, and proportionate trial management. The **ICH E6 (R2) (Good Clinical Practice guidelines)** [29] **will be updated by** ICH E6 (R3), which is still in progress, taking into consideration features such as overarching principles, interventional CTs, and non-traditional interventional CTs. Reference HAs are ICH members, except TGA, so they implement these guidelines. However, most LATAM countries have not formally joined the ICH [30], so implementation of guidelines is not mandatory. This lack of compromise hinders adoption of all new concepts described in these guidelines.

### Overall Discussion and Conclusions

Some HAs are already implementing changes to regulations or publishing guidance to consider new drug development designs and alternative registration procedures, although they are progressing at a different speed [31–33]. Even in the case of reference HAs (FDA, EMA, TGA, Health Canada, PMDA, and Swissmedic), the stages for implementation and acceptance of these novelties are not homogeneous. For LATAM countries, this situation is still more delayed. Therefore, we considered that an analysis of LATAM regulations and their comparison to reference HAs was necessary to determine measures to identify gaps and propose measures to close them. In this review and analysis, we have found a lack of acceptance of alternative registration pathways and innovative CT designs which motivates the call to LATAM stakeholders to follow the steps that reference HAs have already started and to adopt more simple and flexible procedures. One reason that hampers the implementation of these new procedures is the **lack of a regular update** of the drug registration and clinical research requirements in LATAM countries. More frequent reviews and updates of the regulations will facilitate the necessary changes for adopting new CT designs and more agile marketing approvals for a new drug or indication. In addition, implementing measures such as enhancing the adoption of the reliance pathway or **accepting** non-traditional CT phases as sufficient for registration would facilitate alignment with the current setting and modernization. In this sense, FDA is the most advanced agency of the reference HAs, so the key point could be to strengthen its role as a reference agency in these countries. In this way, by simplifying requirements and modernizing the drug development model, new drugs that aim to cover unmet medical needs will be able to reach patients earlier in LATAM countries.

In summary, regulations in LATAM countries must be changed and adapted to the newest regulatory standards to account for novelties and efficiently provide innovative drugs to patients. The path to be followed is adopting regulations from reference HAs, especially the FDA and EMA, as well as international guidelines (ICH). The key points to develop are included in Table 4.

**Table 4:** Key points to be developed in Latin America countries to adapt their regulations to improve the approval of innovative drugs

More frequent updates to local regulations to implement the necessary changes faster according to new needs
Adoption of alternative registration pathways to speed up the marketing authorization process. Mainly they could be: - reliance pathway, which facilitate regulatory decisions, - expedited regulatory pathway, which are focused on drugs addressing unmet medical needs
Promoting early HA—sponsor meetings
More flexible regulations that adopt concept of master protocols (umbrella, basket, platform), adaptive designs, enrichment strategies, and decentralized CTs, or at least not posing roadblocks to these
Elimination of the requirement of traditional procedures to reach phase III before drug approval
Accepting operational changes to the conduct of CTs, such as the use of decentralized CT elements

*CT* clinical trial, *HA* health authority

## Data Availability

The data that support the findings of this study are available from the corresponding author upon reasonable request.
